# The Evaluation of Asymmetry in Isokinetic and Electromyographic Activity (sEMG) of the Knee Flexor and Extensor Muscles in Football Players after ACL Rupture Reconstruction and in the Athletes following Mild Lower-Limb Injuries

**DOI:** 10.3390/jcm12031144

**Published:** 2023-02-01

**Authors:** Łukasz Oleksy, Anna Mika, Iwona Sulowska-Daszyk, Renata Kielnar, Zofia Dzięcioł-Anikiej, Joanna Zyznawska, Olga Adamska, Artur Stolarczyk

**Affiliations:** 1 Faculty of Health Sciences, Department of Physiotherapy, Jagiellonian University Medical College Krakow, 31-008 Krakow, Poland; 2 Oleksy Medical & Sports Sciences, 37-100 Łańcut, Poland; 3 Institute of Clinical Rehabilitation, University of Physical Education in Kraków, al. Jana Pawła II 78, 31-571 Kraków, Poland; 4 Institute of Health Sciences, Medical College of Rzeszów University, 35-315 Rzeszów, Poland; 5 Department of Rehabilitation, Medical University of Bialystok, 24A M. Skłodowskiej-Curie St, 15-276 Bialystok, Poland; 6 Department of Orthopaedics and Rehabilitation, Medical Faculty, Medical University of Warsaw, 02-091 Warsaw, Poland

**Keywords:** isokinetic, sEMG, muscle, symmetry, fatigue, ACL, football players

## Abstract

This study was aimed at evaluating knee stabilizer (quadriceps and hamstring) muscle strength and the medio-lateral symmetry of hamstring fatigue in football players after ACL reconstruction and with mild lower extremity injuries. The study comprised 65 professional football players who were divided into three groups: Group 1 (n = 24; 22.7 ± 3.6 years; 175 ± 4 cm; 77.3 ± 7.6 kg) after ACL reconstruction, Group 2 (n = 21; 20.5 ± 3.7 years; 177 ± 6 cm; 74.3 ± 9.1 kg) with mild lower-limb injuries (grade 1 muscle strains) and Group 3 (n = 20; 23.1 ± 2.8 years; 178 ± 6 cm; 75.8 ± 8.8 kg) without injuries in the past 3 years. The concentric isokinetic test (10 knee flexions and extensions at 60, 180 and 300°/s with a 30 s interval for rest) was performed on both limbs. Fatigue symmetry between the medial and lateral hamstrings was measured with sEMG during 60 s of isometric contractions. In comparison to the other groups, the injured leg demonstrated significantly lower values of peak torque for the quadriceps (G1–G2 = 48%, 38%, 14%; G1–G3 = 49%, 25%, 14%) and hamstring muscles (G1–G2 = 36%, 35%, 18%; G1–G3 = 64%, 28%, 17%) as well as lower values of hamstring muscle work (G1–G2 = 262 J, 157 J; G1–G3 = 219 J, 179 J) and power (G1–G2 = 34 W; 11 W; G1–G3 = 29 W, 12 W). No significant differences were noted in strength between Groups 2 and 3. The significantly higher fatigue of the BF compared to the SEM muscle was seen in Group 1 for the involved (mean difference = 0.12) and uninvolved limbs (mean difference = −0.10), but in Group 2, a non-significant trend towards asymmetry was also noted. No asymmetry in hamstring muscle fatigue was determined in Group 3. The results of our study allow us to indicate that active football players who previously met the RTS criteria, had deficits in lower-limb muscle performance 2–3 years after reconstruction, which could lead to ACL re-injury. This observation is potentially of importance because these deficits may not be subjectively reported by such athletes and also may not be visible in regular orthopedic and physiotherapeutic assessment.

## 1. Introduction 

Anterior cruciate ligament (ACL) injury causes an acute loss of function and prolonged absence from sports activities [1,2]. It has been reported that after an ACL rupture, 81% of patients return to some kind of sport, 65% return to their pre-injury level of sport, while only 55% return to competitive sports [3]. Moreover, during the 5 years following ACL reconstruction, 6–25% of athletes sustain ACL re-injury (graft rupture) and 2–20% suffer from contralateral ACL injury [4,5,6]. Post ACL reconstruction, athletes are frequently permitted to perform sport only on the basis of orthopedic examination or selective functional tests. However, this kind of assessment may not be capable of detecting all motor deficits that occur after such a serious trauma, especially after a prolonged period following reconstruction. Those undetected deficits are associated with the occurrence of compensation and pathological movement patterns leading to further tissue overloads and often, to ACL re-injury [3,4,5]. Therefore, there is a need to verify the method of muscle performance evaluation after ACL reconstruction. 

Isokinetic muscle strength is frequently measured after ACL reconstruction [7]. Most often, the clinical conclusion is based on the limb symmetry index of the injured and uninjured legs (LSI), and hamstring to quadricep (H/Q) ratio of the injured leg [8,9]. Nonetheless, it has been suggested that the LSI frequently overestimates knee function and may be related to ACL re-injury [9,10]. Kyritsis et al. [3] have suggested that sufficient hamstring strength and appropriate balance between hamstring and quadricep muscle strength are important objectives of ACL rehabilitation. As was reported by Kim et al. [11], in patients with ACL rupture, a strength decrease was observed in both quadriceps and hamstring muscles, with the decrease in quadriceps being three-fold greater [12]. Moreover, Grindem et al. [13] have indicated that after ACL reconstruction, deficits in lower-limb muscle function are observed even more than 4 years later. Thus, there is a need to comprehensively evaluate lower-limb muscle strength and determine if any deficits and/or asymmetries are present which may serve as risk factors of re-injury. This is also the case in ACL rupture after mild injuries.

It has been reported that the medial and lateral hamstrings play different roles in the coronal plane control of the knee joint; therefore, individual hamstring muscle injuries may have a specific influence on ACL strain [2,14]. The biceps femoris (BF) is the hamstring muscle most commonly exposed to strain and overuse-related injuries [15]. It has been reported as a factor contributing to increased ACL loading [16]. The insufficiency of BF performance has also been linked to deficits in neuromuscular function of the semimembranosus (SM) and semitendinosus (ST), which may further potentially increase the risk of ACL injury [14,15]. It has also been suggested that changes in lateral-to-medial hamstring activation ratio may be specific to injurious activation patterns [14], but there is a lack of studies in which alterations in these muscles’ electromyographic activity (sEMG) has been evaluated in athletes after ACL reconstruction. In this study, research on the above-mentioned issue has been undertaken for the first time and we have hypothesized that the assessment of asymmetry regarding medial and lateral hamstring muscle fatigue may be a sensitive method for evaluating muscular deficits remaining after ACL reconstruction and/or after mild lower-limb injuries. Therefore, it seems particularly significant that the hamstring muscles should be carefully assessed, especially in football players in whom hamstring weakness frequently occurs due to overloading or acute injuries. 

Consequently, the aim of this work was to assess quadricep and hamstring muscle strength and hamstring muscle fatigue in football players after ACL reconstruction and in those suffering from mild lower-limbs injuries. An isokinetic evaluation of knee flexor and extensor muscle strength as well as an electromyographic assessment of hamstring muscle fatigue were conducted in order to verify whether active football players who meet the return-to-sport (RTS) criteria have deficits in lower-limb muscle performance, which may further lead to re-injury. As a secondary objective of this study, the symmetry of lateral and medial hamstring muscle fatigue was assessed for the first time as changes in electromyographic activity and a new method for muscle imbalance detection was proposed, which may be useful in assessments following ACL reconstruction.

## 2. Materials and Methods

### 2.1. Participants

Football players from professional, regional teams were recruited for this study (Table 1). The participants were divided into 3 groups:

Group 1 (n = 24)—football players after ACL reconstruction (involved leg—after ACL reconstruction, uninvolved leg—contralateral limb without ACL injury);

Group 2 (n = 21)—football players after mild lower-limb injury (grade 1 muscle strains) during previous 2–3 years (involved leg—after mild injury, uninvolved leg—contralateral limb without injury);

Group 3 (n = 20)—football players without injuries within the past 3 years (control group) (left limb equivalent of the involved limb and right limb equivalent of the uninvolved limb; for isokinetic variables, values from the right and left leg were pooled and the mean value was calculated as a reference). 

The inclusion criteria for subjects following ACL reconstruction were: regular football training; first, unilateral ACL rupture and reconstruction 2–3 years before the study; and no additional injuries to the contralateral leg. Among 24 football players, 11 patella tendon autografts and 13 hamstring tendon autografts were used during ACL reconstruction. According to the club’s medical documentation, all football players underwent a full cycle of rehabilitation which lasted 9–13 months prior to RTS. All subjects post ACL reconstruction had previously passed the RTS, which included: standard orthopedic tests, manual tests performed by a physiotherapist, muscle strength evaluation and hop tests.

For Group 2, the inclusion criteria comprised: clearance to play after grade 1 lower-limb muscle injury according to Grassi et al. [17]; and no other lower or upper limb and trunk injuries during the 3 years prior to the study. The players who had grade 1 muscle strains recorded in the club’s medical documentation qualified for Group 2. The inclusion criteria into Group 3 was the lack of any lower or upper limb and trunk injuries in the past. Group 3, without injuries, included footballers who did not have any injuries recorded in the club’s medical documentation and without self-reported injuries within the previous 3 years.

All football players were informed about the research protocol and gave their written informed consent to participate in the study. Approval of the Ethical Committee at Regional Medical Chamber in Kraków was obtained for the research (16/KBL/OIL/2016). All procedures were performed in accordance with the 1964 Declaration of Helsinki and its later amendments.

### 2.2. Procedures

#### 2.2.1. Isokinetic Test

Measurements were performed using an isokinetic dynamometer (System 4, Biodex Medical Systems, Shirley, NY, USA) in a seated position with the lower limb flexed in the hip joint to 90° and the knee axis of rotation concordant with the anatomical axis of the joint. Total range of motion (ROM) was set from full extension to full flexion of the knee joint. The movable arm of the dynamometer was fixed at 1/3 of the distal end of the tibia. Isokinetic testing in full concentric mode was performed on the quadriceps and hamstring muscles of both legs. The tests consisted of 10 maximum isokinetic flexions and extensions in the knee joint at each of the 3 angular velocities: 60°/s, 180°/s and 300°/s, with a 30 s interval for rest between them. The following variables were analyzed: peak torque/body mass, average power and total work for flexion and extension, and hamstring to quadricep peak torque ratio (H/Q ratio). The result was the mean value of 10 contractions for each angular velocity. As previously reported, the reliability of peak torque was good and ICC ranged between 0.85 and 0.98 for knee extension and 0.88 and 0.97 for knee flexion [18,19]. 

#### 2.2.2. SEMG Measurements 

The bioelectrical activity of the medial (biceps femoris—BF) and lateral (semimembranosus and semitendinosus—SEM) hamstring muscles was recorded according to SENIAM guidelines [20,21]. The skin and surface electrodes (Ag/AgCl) (Sorimex, Toruń, Poland) were cleaned with alcohol. At a 2 cm center-to-center distance, they were attached along the direction of the muscle fibers on the lateral and medial hamstring bellies. The signal was registered with 16-bit accuracy and at a sampling rate of 1500 Hz using the Noraxon G2 TeleMyo 2400 unit (Noraxon, Scottsdale, AZ, USA). The EMG signals were filtered with Butterworth high-pass (cutoff frequency of 10 Hz) and low-pass filters (cutoff frequency of 500 Hz). The sEMG signal from the evaluated muscles was measured during 60 s of isometric contraction. The measurement was performed in a prone position with the knee flexed to 60°; against resistance. The data were analyzed using MyoResearch XP software (Noraxon, Scottsdale, AZ, USA). Fatigue-related changes as mean frequency shift were calculated from the sEMG signal via the FFT (Fast Fourier Transform) [20,22]. The unfiltered RAW sEMG signal was analyzed using step-wise FFT in 1000 ms increments over 60 s static contractions. Mean frequency (Hz) was calculated for each window and the regression line (slope) was computed. A more negative slope value meant greater fatigue of the tested muscle [22,23]. 

#### 2.2.3. Statistical Analysis

Statistical analysis was performed using STATISTICA 12.0 Pl software (StatSoft, Poland). The Shapiro–Wilk test was conducted to assess data normality. The paired *t*-test was used to determine the differences in muscle force and muscle fatigue variables between the involved and uninvolved limbs, between the BF and SEM muscles and between graft types. Pearson’s correlation coefficient was calculated between rehabilitation time as well as strength and fatigue variables. The ANOVA test for independent samples was used to determine the significance of differences in the measurement variables across the 3 study groups. When a significant main effect was detected, Tukey’s post hoc test was performed. Due to the high amount of data, we decided to present results separately for within-group and between-group comparisons, but not to use mixed ANOVA with the interaction effects. This approach allowed us to present results in a clear and more readable way. However, during work on the manuscript, we performed the statistical analysis using mixed ANOVA and obtained the same results. The effect size was calculated using Cohen’s d and interpreted as small (0.2–0.3), medium (0.5) or large (>0.8) [24]. Differences were considered statistically significant if the level of the test probability was lower than the assumed level of significance (*p* < 0.05).

## 3. Results

### 3.1. Isokinetic Test

In Group 1, at 60°/s, in the involved leg, a significantly lower torque and power of the quadriceps and hamstring muscles were observed in comparison to the uninvolved leg (Table 2). Furthermore, in Group 1, at 180 °/s, significant differences were noted between the involved and uninvolved legs in torque, work and power of the quadriceps and hamstring muscles (Table 2). There were no significant differences between the legs in Group 2 (Table 2). 

In comparison to Group 2 and 3, the football players from Group 1 obtained significantly lower values of peak torque in the injured leg of both the quadriceps and hamstrings at all three angular velocities (Table 3). The average power of the hamstrings at 60°/s in the injured leg for Group 1 was significantly lower than in other groups. The work of the hamstring muscles was also lower and the difference was not significant, but with an effect size of ES = 0.43–0.75 (Table 3). Moreover, subjects in Group 1 demonstrated significantly lower values of hamstring muscle work and power at 180°/s compared to Group 2 and Group 3 (Table 3). There were no significant differences at 180°/s between Groups 2 and 3 (Table 3). At 300°/s, the hamstring muscle of the involved leg demonstrated non-significantly lower values only at work, but with a relatively strong effect size (ES = 0.47–0.65) (Table 3).

For the uninvolved leg in Group 1, compared to Group 2 at 180°/s and 300°/s, significantly lower values of quadricep and hamstring peak torque, as well as a lower H/Q ratio at 300°/s, were observed (Table 4). Additionally, between Group 1 and 2, for the remaining groups at all three angular velocities, non-significantly lower values were noted for quadricep and hamstring torque, work and power, but with an effect size totaling ES = 0.4–0.92 (Table 4).

### 3.2. SEMG Measurements

In both legs (injured and uninjured), the football players from Group 1 demonstrated significantly higher fatigue for the BF muscle compared to the SEM. When comparing BF muscle activity level between legs, a trend indicating higher fatigue was noted in the injured limb compared to that in the uninjured, but the differences were not significant (*p* > 0.05). The effect size was ES = 0.55. A similarly non-significant trend was noted for the SEM muscles: *p* > 0.05; ES = 0.35 (Table 5). In Group 2, among athletes following mild injuries, the pattern of asymmetric muscle activity between limbs (higher fatigue in the BF than in the SEM and higher fatigue in injured leg) was similar to Group 1, but differences between the BF and SEM muscles as well as between limbs were not significant (*p* > 0.05); however, the effect size totaled ES = 0.26–0.46 (Table 5). Among football players without injuries (Group 3), no significant asymmetries were observed either between the BF and SEM or the limbs (*p* > 0.05) (Table 5).

### 3.3. Influence of Graft Type on MUSCLE Strength and Fatigue in Subjects after ACL Reconstruction

We did not observe any significant influence of graft type on any of the evaluated variables (Table 6).

### 3.4. Influence of Time of Rehabilitation on Muscle Strength and Fatigue in Subjects after ACL Reconstruction

We did not observe any significant relationships between the time of rehabilitation (time between ACL reconstructive surgery and RTS) and knee extension, flexion muscle strength or the BF and SEM muscle fatigue variables (Table 7).

## 4. Discussion

The most important information derived from this study is that athletes following ACL reconstruction demonstrated significant asymmetry in the fatigue of the lateral and medial hamstring muscles. The significantly higher fatigue of the BF muscle compared to the SEM was only present in this group, but in footballers after mild injuries, a non-significant trend towards asymmetry was noted. Additionally, no asymmetry was observed for hamstring muscle fatigue in the control group. Moreover, football players post ACL reconstruction in the injured leg exhibited significantly lower values of peak torque in both the quadriceps and hamstrings at all three angular velocities compared to the two remaining groups. They also demonstrated significantly lower values for hamstring muscle work and power. What is important is that muscle strength in footballers post mild lower-limb injuries and those without injuries did not differ significantly, indicating that their lower-limb muscles were similarly strong. Therefore, we may suggest that the lower quadricep and, mainly, the lower hamstring muscle strength and asymmetry in lateral as well as medial hamstring muscle fatigue is specific for athletes after ACL reconstruction. This is visible for a long period after surgery, which could potentially lead to ACL re-injury. This observation may be important because this muscle strength asymmetry could not be subjectively reported by such athletes or be visible in regular orthopedic or physiotherapeutic assessment.

The majority of athletes do not successfully return to their pre-injury sports level, mainly because they do not regain pre-injury muscle function and they are afraid of re-injury [25,26]. In athletes following ACL reconstruction, muscle strength deficits could be observed even 2 years following reconstruction [10,27,28,29,30]. It has been reported that experiencing a second ACL injury reached 49%, which may suggest the inadequacy of current criteria used to determine an athlete’s readiness to return to sport [10].

An isokinetic strength evaluation is frequently performed in patients after ACL reconstruction; however, studies vary in methodological quality and there is no standardized protocol that could comprehensively diagnose existing deficits in muscle strength, work or power [7]. The isokinetic variables most frequently used in the assessment of muscle strength post ACL reconstruction are the LSI and H/Q ratio of the injured leg [7,8,9]. They also have commonly been used as part of the RTS criteria [7]. In some studies, it has been reported that athletes, before returning to sport, should reach a value >90% of LSI in lower-limb muscle strength [4], while other authors have indicated that an LSI value of >95% is required [31]. Undheim et al. [7] suggested that LSI values should be between >70–90%. Nevertheless, it has also been insinuated that this approach may involve some weaknesses; thus, using the uninvolved limb as a reference may not be optimal [3,10]. Some authors have noted that the LSI of muscle strength may overestimate knee function after ACL reconstruction, and therefore may be related to an increased risk of a second ACL injury [10]. This suggestion is also supported by our results. As was observed in our study, subjects following ACL reconstruction, compared to the remaining two groups of athletes, had lower strength values of the quadriceps and hamstring muscles in the uninjured leg as well. However, a decreased strength in Group 1 was noted at 180°/s and 300°/s, and the reduction was smaller than that noted in the injured leg. However, this observation confirmed that LSI should be considered with caution, especially in subjects post ACL reconstruction. The lower values of muscle strength observed in both legs may be related to an overestimation of knee function by LSI as was suggested by Wellsandt et al. [10] and also observed in this study. Therefore, if both legs are weaker, LSI used during RTS assessment overestimates knee function, and athletes who do not meet the RTS criteria may be classified as healthy and ready to play. Moreover, we did not observe significant differences between limbs in athletes following mild injuries; thus, LSI may overestimate real functional status and the readiness to return to sport in these footballers as well.

In numerous studies, the effect of strain generated by the quadriceps and hamstrings on the ACL during knee motion has been analyzed [32,33,34,35]. These authors reported that isolated and increased quadriceps contraction significantly loads the ACL, but simultaneous hamstring contraction reduces this ACL strain [32,33,36,37]. The hamstring muscles act as agonists to the ACL by resisting the anterior tibial displacement resulting from quadricep muscle forces on the knee [35,36,37,38]. Thus, the weakness of hamstring strength and disturbed H/Q ratio are often reported as risk factors for ACL and/or graft rupture [39,40]. A normal H/Q ratio should be between 0.5 and 0.8 [3,41]. It has been underlined by some authors that sufficient hamstring strength and, consequently, appropriate balance between the hamstrings and quadriceps are an important objective of ACL rehabilitation [3]. In our study, it has been shown that football players exhibited significantly lower values of peak torque regarding both the quadriceps and hamstrings at all three angular velocities in the injured leg compared to the remaining two groups. They also demonstrated significantly lower values of hamstring muscle work and power at 60°/s and 180°/s. Additionally, in the non-involved leg in Group 1, at 180°/s and 300°/s, significantly lower values of H/Q ratio were observed in comparison to the other two groups. What is important is that muscle strength between footballers after mild lower-limb injuries and in those without injuries did not differ significantly, indicating that their lower-limb muscles were similarly strong. Therefore, we may suggest that lower quadricep and, mainly, lower hamstring muscle strength is specific for athletes after ACL reconstruction, even if they had earlier passed an RTS assessment and were cleared to play. 

A relationship between previous hamstring injury and subsequent ACL rupture has been indicated by some authors [14,42]. Due to the fact that hamstring contraction by resisting anterior translation and internal rotation of the tibia relative to the femur may reduce ACL strain, any weakness or neuromuscular dysfunction of the hamstring muscles may lead to an increased risk of ACL injury [36,37,43,44,45]. In our study, football players following ACL reconstruction demonstrated significantly lower values of hamstring muscle work and power in comparison to the remaining two groups. Therefore, we have suggested that the hamstring insufficiency after ACL reconstruction may also be an important factor increasing ACL graft rupture. Another significant issue in the evaluation of motor deficits post ACL reconstruction are the conditions under which testing is performed. Thomee et al. [27] have noted that 1 year after ACL reconstruction, approximately 70% of the evaluated athletes demonstrated motor deficits during testing in fatigued conditions, despite having an LSI value of >90% in the hop test when they were not fatigued. Jordan et al. [46] have also reported increased hamstring activity in the limb after ACL reconstruction after muscle fatigue compared to the contralateral one, and suggested this hamstring behavior as a ACL protective strategy. 

It has further been stated that hamstring injury can lead to general alterations in sagittal knee joint function, but the medial and lateral hamstrings have different roles in knee control at the level of the coronal plane [2]. The lateral hamstrings flex, externally rotate and abduct the tibia, reducing ACL strain during knee flexion; however, the medial hamstrings flex, internally rotate and adduct the tibia [2]. As such, the injury to specific hamstring muscles may have a direct influence on the potential increase in ACL rupture risk [2,14]. It has been reported that the BF insufficiency induced by increasing the varus knee force applied via the medial hamstrings increased the likelihood of a knee valgus kinematic profile, also augmenting the risk of ACL injury [15,47]. The BF is the hamstring muscle most commonly affected by strain, which has been reported as a factor that increases loading on the ACL [15,16]. Therefore, if the BF weakness and/or overuse leads to an increased load on the ACL, the asymmetry between the BF and SEM regarding fatigue observed in our study may indicate a potentially increased risk of ACL graft rupture in footballers after ACL reconstruction. This higher BF fatigue probably reduces the lateral-to-medial activation ratio within the hamstring muscle. Greater fatigue of the BF may cause insufficiency of this muscle in the resistance to anterior tibial translation and an increase in ACL load, as well as failure to laterally stabilize the knee in the frontal plane. Thus, it may be considered that any alterations in fatigue symmetry between the lateral and medial hamstrings could be considered as factors leading to an increased risk of primary ACL as well as graft rupture. There are no existing studies on the evaluation of hamstring medial and lateral fatigue asymmetry. As a result, our work is the first in which research on this issue has been undertaken. This assessment seems very important during rehabilitation and also at the time of return to sport.

The medial hamstrings contribute much less to strain reduction in the ACL; hence, appropriate balance between SEM and BF performance is crucial in ACL graft rupture [2]. It has been noted that in pivoting sports with fatigued and not-well-controlled lateral hamstring muscles, the risk of graft rupture is very high [2,7,46]. Consequently, we suggest that selective and individualized training should be applied to each part–the medial and lateral hamstring muscles—to help better control loadings on the ACL. What is more, in the athletes after mild injuries observed in our study, the pattern of asymmetric muscle activity between limbs (higher fatigue in the BF than in the SEM and higher fatigue in the injured leg) was similar as in athletes post ACL reconstruction, but differences between the BF and SEM muscles, as well as between limbs, were not significant. On the other hand, in football players without injuries, no asymmetries were observed either between the BF and SEM or between the examined limbs. Since the hamstring muscles are double-jointed muscles, acting in three planes, it can be assumed that, to some extent, they are active during each movement performed by the lower limb. Therefore, our observations may indicate that any injury to the lower limbs, even a mild one, may lead to hamstring overload.

Patellar tendon autografts, including hamstring tendon autografts, are commonly used during ACL reconstruction [48,49]. In some studies, outcomes have been compared between grafts, ending in mixed findings [49,50]. It has been reported that hamstring tendon grafts may produce deficits in knee flexion strength [48,50] but others indicated that patients with patellar tendon autografts have greater deficits in extensor muscle strength [27]. Clagg et al. [49] found that at the time of return to sport, subjects after ACL reconstruction had lower anterior reach distance in both the involved and uninvolved limbs, compared to the controls. Furthermore, those with patellar tendon grafts had a lower posterolateral reach distance compared to those with hamstring grafts, noting no differences in the other reach directions. Plisky et al. [51] and Butler et al. [52] also reported similar results regarding decreased anterior knee reach distance. In our study, no differences were observed between graft types. Other authors have reported muscle strength and reach distance deficits visible at the time of RTS testing, but we performed the tests at a time much later, following ACL reconstructive surgery. It was also reported by Abourezk et al. [48] that hamstring strength asymmetry is common even 3 years after ACL reconstruction with a hamstring tendon autograft that may affect knee mechanics. In our research, 2–3 years after ACL reconstruction, no significant differences were observed between graft types. This discrepancy may be due to the fact that Abourezk et al. [48] assessed isometric hamstring muscle strength, but in our study, the isokinetic mode was used, which was more functional. On the other hand, Ageberg et al. [53], 2 to 5 years after the ACL rupture, did not find any differences between patients who had undergone surgical reconstruction and rehabilitation and those who had been treated only with rehabilitation. Their results may suggest that the graft type may not be a critical factor in the presence of strength deficits, and also that the surgery itself does not always provide better results than conservative treatment. Moreover, we did not observe any significant relationship between muscle strength and time of rehabilitation after ACL reconstruction. Therefore, it is unclear whether the graft choice and time of rehabilitation significantly influences the recovery of muscle performance in patients undergoing ACL reconstruction.

This study also has some limitations which should be addressed. We have evaluated only football players. Consequently, future research is required. It should include athletes from other disciplines. Moreover, the study design was observational and the subjects were assessed only once. Thus, longitudinal monitoring of muscle strength and bioelectrical activity would be of interest.

## 5. Conclusions

Our results have allowed us to indicate that athletes after ACL reconstruction demonstrated significant asymmetry in the fatigue of the lateral and medial hamstring muscles. However, a significantly higher fatigue of the BF muscle compared to SEM was present only in this group, but a similar, non-significant pattern of asymmetric hamstring activity was also noted in footballers following mild injuries. What is important is that the footballers without injuries demonstrated symmetrical lateral and medial hamstring fatigue. This observation may suggest that any kind of lower-limb injury may influence the knee flexors and lead to muscle performance alterations. The fact that in footballers post ACL reconstruction, with the effort size being identical, the BF muscle became fatigued faster than the SEM muscles, should be considered as an important sign of knee flexor imbalance, which may be a risk factor for future graft rupture. This lateral to medial hamstring asymmetry co-existed with significant strength deficits in both the injured and uninjured limbs compared to footballers post mild injuries and to the control group. Moreover, muscle strength in footballers after mild lower-limb injuries and in those without them did not differ significantly, indicating that their lower-limb muscles were similarly strong. Therefore, we may suggest that lower quadricep and, mainly, lower hamstring muscle strength, as well as asymmetry in lateral and medial hamstring muscle fatigue, is specific for athletes following ACL reconstruction. The results achieved in our study allow us to indicate that active football players who previously meet the RTS criteria 2–3 years after reconstruction have deficits in lower-limb muscle performance, which may lead to ACL re-injury. This observation may be important because these deficits might not have been subjectively reported by such athletes and also may not have been visible in regular orthopedic and physiotherapeutic assessment. We have also indicated that muscle fatigue, especially when it causes asymmetry, should be carefully evaluated and monitored during early rehabilitation, and later, after RTS. 

## Figures and Tables

**Table 1:** Subjects’ characteristics.

	Group 1	Group 2	Group 3	*p*
Number of subjects (n)	24	21	20	0.45
Height (cm)	175 ± 4	177 ± 6	178 ± 6	0.86
Weight (kg)	77.3 ± 7.6	74.3 ± 9.1	75.8 ± 8.8	0.88
Age	22.7 ± 3.6	20.5 ± 3.7	23.1 ± 2.8	0.95

*p*—*p* value between groups.

**Table 2:** Comparison of knee extension and flexion muscle strength variables between limbs within each study group.

Outcome Measure	Group 1				Group 2				Group 3			
	Involved	Uninvolved	*p*	ES	Involved	Uninvolved	*p*	ES	Right	Left	*p*	ES
Peak torque extension 60°/s [%]	226 ± 61	251 ± 44	**0.03**	**0.47**	274 ± 47	282 ± 57	0.38	0.15	289 ± 80	260 ± 97	0.10	0.32
Peak torque flexion 60°/s [%]	121 ± 25	131 ± 20	**0.01**	**0.44**	157 ±31	155 ± 31	0.68	0.06	183 ± 140	187 ± 158	0.44	0.02
Average power extension 60°/s [W]	107 ± 30	121 ± 27	**0.03**	**0.49**	120 ± 26	124 ± 28	0.46	0.14	121 ± 31	118 ± 31	0.47	0.09
Average power flexion 60°/s [W]	60 ± 16	69 ± 17	**0.01**	**1.02**	77 ± 17	78 ± 18	0.74	0.05	70 ± 18	75 ± 20	0.09	0.26
Total work extension 60°/s [J]	931 ± 271	1018 ± 211	0.12	0.35	968 ± 209	968 ± 236	0.98	0.01	931 ± 250	866 ± 224	0.11	0.27
Total work flexion 60°/s [J]	540 ± 127	579 ± 169	**0.20**	0.26	611 ± 193	645 ± 158	0.44	0.19	543 ± 209	575 ± 169	0.41	0.16
H/Q ratio 60°/s [%]	57 ± 18	51 ± 14	**0.11**	**0.37**	58 ± 11	55 ± 8	0.19	0.31	55 ± 10	59 ± 7	**0.03**	**0.46**
Peak torque extension 180°/s [%]	153 ± 35	169 ± 25	**0.02**	**0.52**	191 ± 34	197 ± 37	0.15	0.16	184 ± 24	172 ± 25	**0.0009**	**0.48**
Peak torque flexion 180°/s [%]	80 ± 17	91 ± 19	**0.0004**	**0.61**	115 ± 21	120 ± 27	0.40	0.20	105 ± 24	111 ± 22	0.08	0.26
Average power extension 180°/s [W]	195 ± 49	216 ± 42	**0.03**	**0.46**	211 ± 45	218 ± 32	0.35	0.17	212 ± 39	203 ± 45	0.17	0.21
Average power flexion 180°/s [W]	88 ± 28	106 ± 37	**0.001**	**0.54**	122 ± 41	124 ± 32	0.80	0.05	115 ± 40	120 ± 38	0.37	0.12
Total work extension 180°/s [J]	1374 ± 339	1501 ± 267	**0.04**	**0.41**	1467 ± 312	1401 ± 382	0.46	0.18	1472 ± 280	1377 ±302	**0.04**	**0.32**
Total work flexion 180°/s [J]	634 ± 226	741 ± 278	**0.004**	**0.42**	896 ± 297	900 ± 230	0.93	0.01	837 ± 282	869 ± 259	0.49	0.11
H/Q ratio 180°/s [%]	56 ± 24	54 ± 10	0.67	0.10	60 ± 8	59 ± 8	0.63	0.12	57 ± 12	65 ± 11	**0.0002**	**0.69**
Peak torque extension 300°/s [%]	117 ± 22	125 ± 15	0.07	0.42	131 ± 24	138 ± 18	0.07	0.32	135 ± 17	127 ± 19	0.07	0.44
Peak torque flexion 300°/s [%]	70 ± 14	75 ± 17	0.07	0.32	88 ± 15	96 ± 15	**0.02**	**0.53**	86 ± 20	88 ± 19	0.65	0.10
Average power extension 300°/s [W]	203 ± 48	215 ± 41	0.14	0.26	208 ± 46	222 ± 35	**0.04**	**0.34**	209 ± 43	197 ± 30	0.08	0.32
Average power flexion 300°/s [W]	96 ± 37	103 ± 41	0.28	0.17	107± 37	119 ± 30	0.07	0.35	107± 39	110 ± 35	0.51	0.08
Total work extension 300°/s [J]	1971 ± 439	2072 ± 397	0.17	0.24	1995 ± 405	2097 ± 323	0.11	0.27	2065 ± 407	1946 ± 319	**0.04**	**0.32**
Total work flexion 300°/s [J]	948 ± 309	1048 ± 399	0.09	0.28	1105 ± 348	1221 ± 309	0.10	0.35	1109 ± 372	1149 ± 365	0.11.	0.10
H/Q ratio 300°/s [%]	62 ± 17	60 ± 11	0.62	0.13	68 ± 11	70 ± 9	0.51	0.19	64 ± 11	69 ± 14	0.39	0.39

*p*-value between right and left side within each group; ES—effect size (Cohen d); values are expressed as mean ± SD.

**Table 3:** Between-group comparison of the involved leg knee extension and flexion muscle strength variables.

Outcome Measure	Group 1			Group 2			Group 3		
	Involved Leg	*p* ^G1–G2^	ES	Involved leg	*p* ^G2–G3^	ES	Control leg	*p* ^G1–G3^	ES
Peak torque extension 60°/s [%]	226 ± 61	**0.03**	**0.88**	274 ± 47	0.73	0.22	275 ± 81	**0.005**	**0.68**
Peak torque flexion 60°/s [%]	121 ± 25	**0.004**	**1.27**	157 ±31	0.57	0.25	185 ± 40	**0.03**	**0.59**
Average power extension 60°/s [W]	107 ± 30	0.43	0.46	120 ± 26	0.61	0.03	120 ± 29	0.49	0.45
Average power flexion 60°/s [W]	60 ± 16	**0.005**	**1.02**	77 ± 17	0.40	0.39	72 ± 18	0.15	0.58
Total work extension 60°/s [J]	931 ± 271	0.37	0.15	968 ± 209	0.51	0.16	899 ± 221	0.47	0.01
Total work flexion 60°/s [J]	540 ± 127	0.33	0.43	611 ± 193	0.71	0.33	559 ± 170	0.39	0.01
H/Q ratio 60°/s [%]	57 ± 18	0.46	0.06	58 ± 11	0.56	0.28	57 ± 8	0.52	0.13
Peak torque extension 180°/s [%]	153 ± 35	**0.0007**	**1.10**	191 ± 34	0.78	0.23	178 ± 24	**0.006**	**0.83**
Peak torque flexion 180°/s [%]	80 ± 17	**0.0001**	**1.83**	115 ± 21	0.25	0.44	108 ± 22	**0.0009**	**1.42**
Average power extension 180°/s [W]	195 ± 49	0.66	0.34	211 ± 45	0.71	0.02	208 ± 39	0.62	0.38
Average power flexion 180°/s [W]	88 ± 28	**0.008**	**0.96**	122 ± 41	0.78	0.17	117 ± 36	0.07	0.78
Total work extension 180°/s [J]	1374 ± 339	0.65	0.28	1467 ± 312	0.44	0.01	1424 ± 274	0.34	0.31
Total work flexion 180°/s [J]	634 ± 226	**0.005**	**0.99**	896 ± 297	0.76	0.20	853 ± 249	**0.04**	**0.92**
H/Q ratio 180°/s [%]	56 ± 24	0.76	0.22	60 ± 8	0.78	0.29	61 ± 10	0.65	0.05
Peak torque extension 300°/s [%]	117 ± 22	0.08	0.60	131 ± 24	0.82	0.19	131 ± 14	**0.02**	**0.75**
Peak torque flexion 300°/s [%]	70 ± 14	**0.002**	**1.24**	88 ± 15	0.99	0.11	87 ± 18	**0.006**	**1.05**
Average power extension 300°/s [W]	203 ± 48	0.99	0.10	208 ± 46	0.97	0.02	203 ± 35	0.98	0.13
Average power flexion 300°/s [W]	96 ± 37	0.92	0.29	107 ± 37	0.78	0.01	108 ± 36	0.89	0.28
Total work extension 300°/s [J]	1971 ± 439	0.67	0.05	1995 ± 405	0.87	0.17	2005 ± 344	0.45	0.22
Total work flexion 300°/s [J]	948 ± 309	0.58	0.47	1105 ±348	0.88	0.01	1127 ± 355	0.67	0.47
H/Q ratio 300°/s [%]	62 ± 17	0.64	0.41	68 ± 11	0.87	0.36	66 ± 11	0.77	0.13

*p*^G1–G2^-value between Group 1 and Group 2 (*p* is the post hoc value of the study group’s main effect); *p*^G2–G3^-value between Group 2 and Group 3 (*p* is the post hoc value of the study group’s main effect); *p*^G1–G3^-value between Group 1 and Group 3 (*p* value is the post hoc value of the study group’s main effect); ES–effect size (Cohen’s d); values are expressed as mean ± SD.

**Table 4:** Between-group comparison of uninvolved leg knee extension and flexion muscle strength variables.

Outcome Measure	Group 1			Group 2			Group 3		
	Uninvolved Leg	*p* ^G1–G2^	ES	Uninvolved Leg	*p* ^G2–G3^	ES	Control leg	*p* ^G1–G3^	ES
Peak torque extension 60°/s [%]	251 ± 44	0.85	0.60	282 ± 57	0.90	0.27	275 ± 81	0.99	0.11
Peak torque flexion 60°/s [%]	131 ± 20	0.89	0.92	155 ± 31	0.87	0.28	185 ± 40	0.82	0.49
Average power extension 60°/s [W]	121 ± 27	0.76	0.10	124 ± 28	0.87	0.20	120 ± 29	0.75	0.10
Average power flexion 60°/s [W]	69 ± 17	0.65	0.51	78 ± 18	0.87	0.15	72 ± 18	0.55	0.32
Total work extension 60°/s [J]	1018 ± 211	0.37	0.22	968 ± 236	0.67	0.44	899 ± 221	0.41	0.55
Total work flexion 60°/s [J]	579 ± 169	0.45	0.40	645 ± 158	0.69	0.42	559 ± 170	0.44	0.02
H/Q ratio 60°/s [%]	51 ± 14	0.56	0.35	55 ± 8	0.88	0.53	57 ± 8	0.34	0.52
Peak torque extension 180°/s [%]	169 ± 25	**0.006**	**0.88**	197 ± 37	**0.02**	**0.79**	178 ± 24	0.24	0.36
Peak torque flexion 180°/s [%]	91 ± 19	**0.0004**	**1.24**	120 ± 27	0.12	0.36	108 ± 22	**0.01**	**0.82**
Average power extension 180°/s [W]	216 ± 42	0.65	0.05	218 ± 32	0.76	0.38	208 ± 39	0.65	0.29
Average power flexion 180°/s [W]	106 ± 37	0.23	0.52	124 ± 32	0.77	0.11	117 ± 36	0.39	0.37
Total work extension 180°/s [J]	1501 ± 267	0.43	0.30	1401 ± 382	0.85	0.06	1424 ± 274	0.23	0.43
Total work flexion 180°/s [J]	741 ± 278	0.13	0.62	900 ± 230	0.67	0.12	853 ± 249	0.28	0.47
H/Q ratio 180°/s [%]	54 ± 10	0.17	0.55	59 ± 8	0.09	0.62	61 ± 10	**0.001**	**0.70**
Peak torque extension 300°/s [%]	125 ± 15	**0.03**	**0.78**	138 ± 18	0.23	0.59	131 ± 14	0.31	0.41
Peak torque flexion 300°/s [%]	75 ± 17	**0.0006**	**1.30**	96 ± 15	0.27	0.46	87 ± 18	0.14	0.68
Average power extension 300°/s [W]	215 ± 41	0.67	0.18	222 ± 35	0.12	0.76	203 ± 35	0.33	0.50
Average power flexion 300°/s [W]	103 ± 41	0.25	0.44	119 ± 30	0.67	0.27	108 ± 36	0.71	0.18
Total work extension 300°/s [J]	2072 ± 397	0.78	0.06	2097 ± 323	0.56	0.47	2005 ± 344	0.43	0.34
Total work flexion 300°/s [J]	1048 ± 399	0.51	0.48	1221 ±309	0.56	0.21	1127 ± 355	0.52	0.26
H/Q ratio 300°/s [%]	60 ± 11	**0.02**	**0.99**	70 ± 9	0.17	0.08	66 ± 11	**0.04**	**0.54**

*p*^G1–G2^-value between Group 1 and Group 2 (*p* is the post hoc value of the study group’s main effect); *p*^G2–G3^-value between Group 2 and Group 3 (*p* is the post hoc value of the study group’s main effect); *p*^G1–G3^-value between Group 1 and Group 3 (*p* is the post hoc value of the study group’s main effect); ES–effect size (Cohen’s d); values are expressed as mean ± SD.

**Table 5:** Comparison of biceps femoris (BF) and semimembranosus-semitendinosus (SEM) muscle fatigue.

Outcome Measure	Side	BF Slope (Hz)	*p* ^#^	ES ^#^	SEM Slope (Hz)	*p* ^#^	ES ^#^	*p* *	ES *
**Group 1**	I(R)	−0.16 ± 0.20 (0.05)	0.14	0.55	−0.04 ± 0.25 (0.05)	0.41	0.35	**0.016**	0.53
	U(L)	−0.05 ± 0.20 (0.04)			0.05 ± 0.26 (0.06)			**0.004**	0.43
**Group 2**	I(R)	−0.12 ± 0.26 (0.06)	0.28	0.29	−0.03 ± 0.41 (0.09)	0.21	0.41	0.38	0.26
	U(L)	−0.03 ± 0.34 (0.07)			0.11 ± 0.25 (0.05)			0.19	0.46
**Group 3**	I(R)	0.04 ± 0.25 (0.05)	0.99	0.27	0.03 ± 0.23 (0.05)	0.48	0.34	0.85	0.04
	U(L)	0.04 ± 0.29 (0.07)			0.11 ± 0.24 (0.05)			0.38	0.26

I(R)—involved (right) side; U(L)—uninvolved (left) side; *p* ^#^-value between involved (right) and uninvolved (left) side within each group; *p* *—value between BF and SEM muscle; ES ^#^—effect size (Cohen’s d) between involved (right) and uninvolved (left) side within each group; ES *—effect size (Cohen’s d) between BF and SEM muscle; values are expressed as mean ± SD (SEM).

**Table 6:** Comparison of knee extension and flexion muscle strength and biceps femoris (BF) and semimembranosus-semitendinosus (SEM) muscle fatigue between subjects with patella tendon autograft and hamstring tendon autograft used during ACL reconstruction.

Outcome Measure	Patella Graft (n = 11)	Hamstring Graft (n = 13)	*p*
	Mean ± SD	Mean ± SD	
Peak torque extension 60°/s [%]	219 ± 51	232 ± 70	0.60
Peak torque flexion 60°/s [%]	117 ± 22	124 ± 27	0.52
Average power extension 60°/s [W]	109 ± 33	106 ± 28	0,78
Average power flexion 60°/s[W]	60 ± 18	60 ± 14	0.97
Total work extension 60°/s [J]	931 ± 294	931 ± 263	0.99
Total work flexion 60°/s [J]	539 ± 156	540 ± 102	0.98
H/Q ratio 60°/s [%]	56 ± 15	58 ± 22	0.74
Peak torque extension 180°/s [%]	152 ± 28	154 ± 41	0.91
Peak torque flexion 180°/s [%]	83 ± 18	78 ± 22	0.46
Average power extension 180°/s [W]	208 ± 48	184 ± 48	0.23
Average power flexion 180°/s [W]	95 ± 24	82 ± 31	0.28
Total work extension 180°/s [J]	1420 ± 332	1335 ± 353	0.55
Total work flexion 180°/s [J]	650 ± 250	621 ± 213	0.75
H/Q ratio 180°/s [%]	56 ± 10	56 ± 32	0.98
Peak torque extension 300°/s [%]	118 ± 18	116 ± 26	0.82
Peak torque flexion 300°/s [%]	74 ± 19	67 ± 17	0.20
Average power extension 300°/s [W]	220 ± 48	190 ± 45	0.12
Average power flexion 300°/s [W]	111 ± 33	83 ± 35	0.07
Total work extension 300°/s [J]	2107 ± 401	1856 ± 453	0.17
Total work flexion 300°/s [J]	1049 ± 232	863 ± 348	0.14
H/Q ratio 300°/s [%]	64 ± 10	61 ± 12	0.68
BF slope (Hz)	−0.15 ± 0.21	−0.18 ± 0.17	0.39
SEM slope (Hz)	−0.05 ± 0.23	−0.03 ± 0.25	0.22

*p*-value between subjects with patella tendon autograft and hamstring tendon autograft.

**Table 7:** Relationship between knee extension and flexion muscle strength and biceps femoris (BF) and semimembranosus-semitendinosus (SEM) muscle fatigue as well as duration of rehabilitation in subjects after ACL reconstruction.

Outcome Measure	Duration of Rehabilitation (Months)	
	r	*p*
Peak torque extension 60°/s [%]	−0.18	0.38
Peak torque flexion 60°/s [%]	−0.31	0.12
Average power extension 60°/s [W]	−0.15	0,46
Average power flexion 60°/s [W]	−0.20	0.33
Total work extension 60°/s [J]	−0.23	0.24
Total work flexion 60°/s [J]	−0.28	0.17
H/Q ratio 60°/s [%]	0.002	0.99
Peak torque extension 180°/s [%]	−0.08	0.68
Peak torque flexion 180°/s [%]	0.19	0.36
Average power extension 180°/s [W]	0.04	0.83
Average power flexion 180°/s [W]	0.17	0.39
Total work extension 180°/s [J]	−0.09	0.65
Total work flexion 180°/s [J]	0.04	0.83
H/Q ratio 180°/s [%]	0.15	0.45
Peak torque extension 300°/s [%]	0.04	0.81
Peak torque flexion 300°/s [%]	0.24	0.24
Average power extension 300°/s [W]	0.13	0.53
Average power flexion 300°/s [W]	0.24	0.23
Total work extension 300°/s [J]	0.05	0.78
Total work flexion 300°/s [J]	0.02	0.18
H/Q ratio 300°/s [%]	0.14	0.50
BF slope (Hz)	0.006	0.97
SEM slope (Hz)	0.10	0.60

r–correlation coefficient; *p*–significance level.

## Data Availability

All data generated or analyzed during this study are included in this published article.
